# Is hospital volume related to quality of hip fracture care? Analysis of 43,538 patients and 68 hospitals from the Dutch Hip Fracture Audit

**DOI:** 10.1007/s00068-022-02205-5

**Published:** 2023-01-21

**Authors:** Franka S. Würdemann, Erik W. van Zwet, Pieta Krijnen, Johannes H. Hegeman, Inger B. Schipper, The Dutch Hip Fracture Audit Group, A. H. Calf, P. W. van Egmond, M. van Eijk, M. van Heijl, M. C. Luyten, B. G. Schutte, S. C. Voeten, A. J. Arends, M. J. Heetveld, M. C. Trappenburg

**Affiliations:** 1 Department of Trauma Surgery, Leiden University Medical Center, Albinusdreef 2, 2333 ZA Leiden, The Netherlands; 2 Dutch Institute for Clinical Auditing, Scientific Bureau, Rijnsburgerweg 10, 2333 AA Leiden, The Netherlands; 3 Department of Biomedical Data Sciences, Leiden University Medical Center, Albinusdreef 2, 2333 ZA Leiden, The Netherlands; 4 Department of Trauma Surgery, Ziekenhuisgroep Twente, Zilvermeeuw 1, 7609 PP Almelo, The Netherlands

**Keywords:** Hospital volume, Hip fracture, Quality of care

## Abstract

**Purpose:**

Evidence for a hospital volume–outcome relationship in hip fracture surgery is inconclusive. This study aimed to analyze the association between hospital volume as a continuous parameter and several processes and outcomes of hip fracture care.

**Methods:**

Adult patients registered in the nationwide Dutch Hip Fracture Audit (DHFA) between 2018 and 2020 were included. The association between annual hospital volume and turnaround times (time on the emergency ward, surgery < 48 h and length of stay), orthogeriatric co-treatment and case-mix adjusted in-hospital and 30 days mortality was evaluated with generalized linear mixed models with random effects for hospital and treatment year. We used a fifth-degree polynomial to allow for nonlinear effects of hospital volume. P-values were adjusted for multiple comparisons using the Bonferoni method.

**Results:**

In total, 43,258 patients from 68 hospitals were included. The median annual hospital volume was 202 patients [range 1–546]. Baseline characteristics did not differ with hospital volume. Provision of orthogeriatric co-treatment improved with higher volumes but decreased at > 367 patients per year (*p* < 0.01). Hospital volume was not significantly associated with mortality outcomes. No evident clinical relation between hospital volume and turnaround times was found.

**Conclusion:**

This is the first study analyzing the effect of hospital volume on hip fracture care, treating volume as a continuous parameter. Mortality and turnaround times showed no clinically relevant association with hospital volume. The provision of orthogeriatric co-treatment, however, increased with increasing volumes up to 367 patients per year, but decreased above this threshold. Future research on the effect of volume on complications and functional outcomes is indicated.

## Introduction

The expected increase in hip fracture incidence due to the aging population underlines the importance of effective and efficient treatment, leading to the best achievable outcomes. [1–3] There is a growing interest in centralization of hip fracture care as treating hip fracture patients in higher-volume hospitals may allow for system-based solutions to minimize operative delay and enable co-management by multidisciplinary teams, thereby improving outcomes while being more cost-effective [4–7].

The hospital volume–outcome relationship has been examined in several surgical fields, for which a positive effect of higher hospital volume was found on mortality, length of stay, costs and readmissions [8]. Almost 90 percent of the studies published on the hospital volume–outcome relationship in orthopedic surgery found a positive effect of higher hospital volumes on outcomes [8]. However, evidence for the relationship between hospital volume and outcomes of hip fracture surgery is inconclusive [9–11].

The varying results in the literature concerning hip fracture surgery outcomes and hospital volume are likely caused by the wide range of cutoff values used to define hospital volume, which is a common problem in volume–outcome analyses [12]. To overcome this problem, the volume–outcome relationship might be studied with hospital volume on a continuous scale [10]. Another drawback of the literature on this topic is that most studies focus primarily on mortality as outcome of care. Other outcomes may also be associated with hospital volume and may therefore be of consequence for setting thresholds or defining the quality of care provided by hospitals [8, 12]. Hence, there is a need for analyses with hospital volume as a continuous parameter that focus on more outcomes of care than mortality only.

This study aimed to analyze the effect of hospital volume as a continuous parameter on several processes in and outcomes of hip fracture care, using data obtained from the nationwide Dutch Hip Fracture Audit in the Netherlands.

## Material and methods

Data for this cohort study were retrieved from the Dutch Hip Fracture Audit (DHFA), a nationwide registry of hip fracture patients in the Netherlands [13]. All registered adult hip fracture patients treated in 68 hospitals between 1-1-2018 and 31-12-2020 were included. Patients with non-operative treatment and patients who suffered a peri-prosthetic or pathological fracture were excluded. DHFA data were supplemented with dates of death from the Dutch Vektis institute, which collects data from health insurance reimbursements [14]. DHFA and Vektis data were joined by a trusted third party using social security numbers.

The outcomes and processes used in the analysis were measured on patient level. Therefore, hospital volume was defined as the number of hip fracture patients treated in a specific hospital and calendar year, and was assigned to every patient treated in that hospital in that particular year.

Two types of outcome measures were studied: (1) hospital process variables measured, including turnaround times [length of stay in the emergency department (ED) in minutes, time to surgery within 48 h and duration of hospital stay in days (HLOS)] and orthogeriatric co-treatment, and (2) outcomes including in-hospital and 30 days mortality.

Evidently incorrect values for process times were excluded from the analyses to avoid bias. These values included entries exceeding 24 h of stay in the ED, 7 days to surgery and 90 days of hospital stay. Orthogeriatric co-treatment was analyzed for patients aged 70 years or older. Orthogeriatric co-treatment was considered present if a geriatrician or internal medicine physician specialized in the elderly either was consulted peri-operatively or was head practitioner or if the patient was treated on a specialized geriatric trauma ward. In the case of a onetime postoperative consultation, orthogeriatric co-treatment was considered absent, as this is not according to the standards of the Dutch Health Care Inspectorate (IGJ).

Case-mix variables included age, sex, fracture side, fracture type, pre-fracture mobility, degree of independence, comorbidity, pre-fracture diagnosis of osteoporosis and risk of malnutrition. Fracture types were defined as undislocated and dislocated femoral neck fractures, trochanteric fractures type AO-A1, AO-A2 and AO-A3, and subtrochanteric fractures [15]. Pre-fracture mobility was based on the Fracture Mobility Score [18]. Pre-fracture degree of independence was based on the KATZ Index of Activities of Daily Living (KATZ-6 ADL) score [16] and categorized as independent (KATZ-6-ADL = 0) or dependent (KATZ-6 ADL ≥ 1). Comorbidity was based on the preoperative American Society of Anesthesiologist physical status classification (ASA score) [17] and categorized as no or mild systemic disease (ASA 1–2) and severe or life-threatening systemic disease (ASA 3–5). The risk of malnutrition was measured during hospital stay using the Short Nutritional Assessment Questionnaire (SNAQ) or the Malnutrition Universal Screening Tool (MUST) and categorized as low (SNAQ = 0 or MUST = 0), medium (SNAQ 1–2 or MUST 1) or high risk (SNAQ ≥ 3, MUST ≥ 2) [18, 19].

### Statistical analysis

Baseline characteristics are reported using descriptive statistics. For reporting patient and treatment characteristics, hospital volume was divided into quartiles representing low, low–mid, mid–high and high annual hospital volume. To determine whether there was an association between hospital volume and outcome measures, mixed-effects regression models were constructed with hospital volume as a predictor. To account for the association between patients treated within the same hospital, we added hospital as a random intercept. To allow for a flexible relation between volume and the dependent variable, we used a polynomial with a degree between 1 and 5, which was determined using Akaike's information criterion [20].

In analyzing mortality as outcome measure, all case-mix factors described above were added as fixed effects. Missing values for case-mix factors were imputed with the median value for age and the mode for the categorical variables.

The effect of hospital volume on outcomes was tested by comparing the fit of models with and without hospital volume. To account for multiple testing, we adjusted the significance level according to Bonferroni; we multiplied all *p* values by the number of tests (i.e., 8) [21]. Adjusted *p* values < 0.05 were regarded statistically significant.

Statistical analysis was performed using R Version 4.0.2 using the ‘lme4’ package for the mixed-effects analysis [22, 23].

## Results

**Fig. 1:**
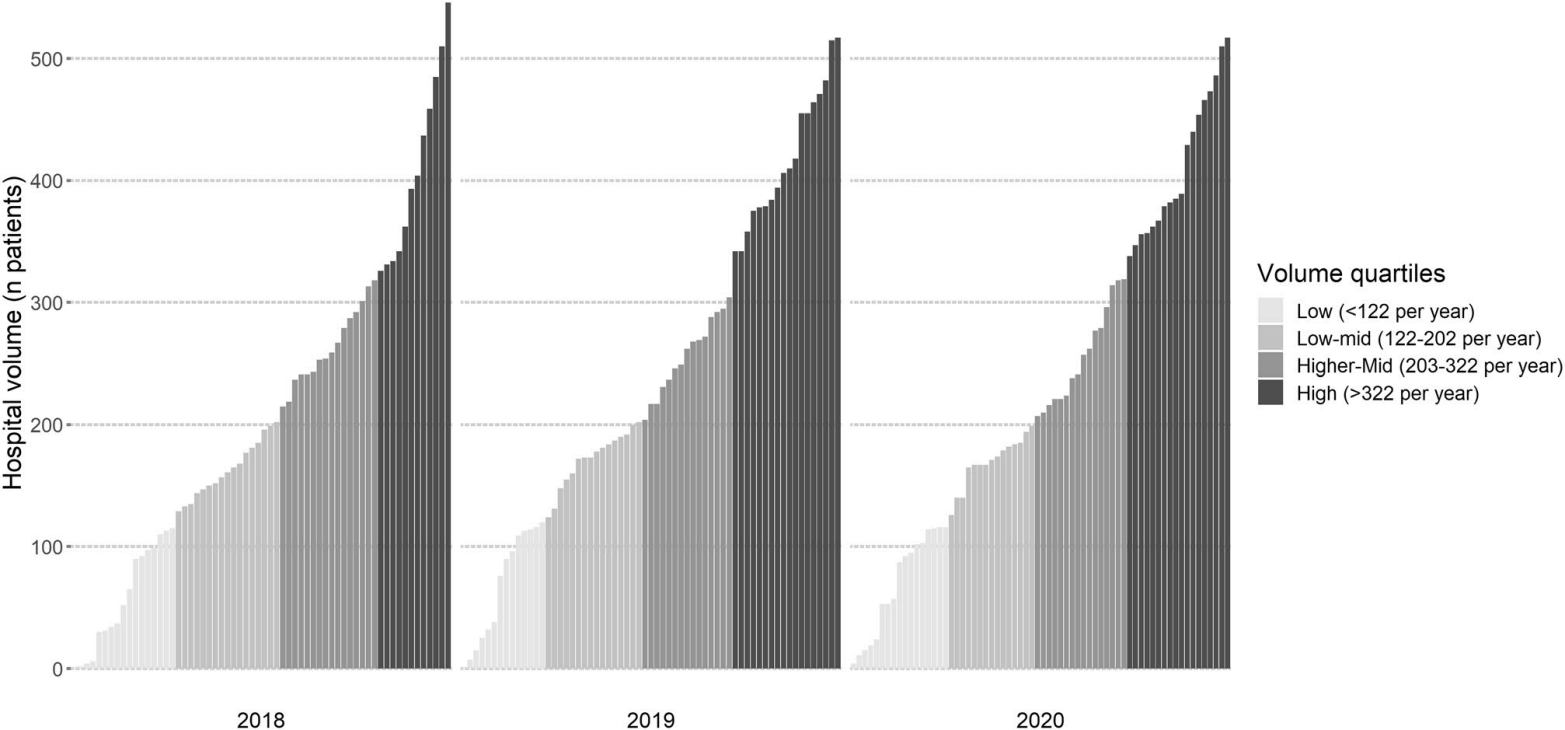
Overview of annual hospital volume of hospitals participating in the Dutch Hip Fracture Audit from 2018 to 2020

Sixty-two hospitals registered data of hip fracture patients in the DHFA in 2018, 63 in 2019 and 68 in 2020. Data of 43,258 operatively treated patients were available for analysis. The median annual registered hospital volume was 202 patients and ranged between 1 and 546. Annual hospital volumes were similar in the three calendar years (Fig. 1). Patient characteristics showed no clinically relevant differences between volume quartile categories (Table 1). Data quality of turnaround times, orthogeriatric co-treatment and mortality was the lowest in low-volume hospitals (Table 1). The registration of these parameters was considered adequate: For none of these parameters, missingness exceeded 10% between 2018 and 2022.

**Table 1:** Patient characteristics and outcomes of patients included in the Dutch Hip Fracture Audit from 2018 to 2020 by annual hospital volume

	Annual hospital volume^a^				
	Low	Low–Mid	Mid–High	High	Total
	*n* = 3106	*n* = 8071	*n* = 12,170	*n* = 19,911	*n* = 43,258
Patient characteristics					
Age (median [IQR])	79 [71, 86]	81 [73, 88]	82 [73, 88]	81 [72, 88]	81 [72, 88]
Missing	0.3	0.3	0.2	0.3	0.3
Female sex (%)	64.6	66.6	67.2	66.0	66.3
Missing	0.0	0.1	0.2	0.2	0.2
Fracture side (%)					
Right	47.2	47.6	48.1	47.7	47.8
Left	52.8	52.2	51.6	51.9	51.9
Bilateral	0.0	0.0	0.1	0.0	0.0
Missing	0.1	0.1	0.2	0.4	0.3
Fracture type (%)					
Femoral neck, undislocated	15.1	15.1	14.6	18.9	16.7
Femoral neck, dislocated	39.9	36.7	38.0	36.2	37.1
Trochanteric type AO-A1	10.8	14.7	12.4	14.0	13.4
Trochanteric type AO-A2	16.2	19.2	19.3	18.4	18.7
Trochanteric type AO-A3	5.6	6.3	6.2	5.3	5.8
Subtrochanteric	4.5	4.1	4.1	3.6	3.9
Unspecified/missing	7.8	3.9	5.3	3.5	4.4
Treatment (%)					
Hemiarthroplasty	29.1	33.5	35.1	36.8	35.1
Total hip arthroplasty	14.3	7.2	4.9	6.5	6.7
Cannulated screws	7.8	5.6	4.7	5.1	5.3
Dynamic or sliding hip screw	13.2	11.5	15.1	13.6	13.6
I ntramedullary nailing	35.6	42.2	40.2	38.0	39.2
Girdle stone	0.0	0.0	0.0	0.1	0.0
Pre-fracture mobility (%)					
Not using any mobility aid	42.8	49.4	45.2	45.3	45.9
Mobile outdoors using 1 mobility aid	6.3	6.7	6.0	6.0	6.1
Mobile outdoors with 2 aids or frame	22.1	31.7	31.2	29.1	29.7
Mobile indoors but never outside without help of others	6.7	5.2	8.5	7.1	7.1
No functional mobility (no use of lower extremities)	2.0	1.2	1.1	3.2	2.2
Missing	20.0	5.8	7.9	9.4	9.0
Dependent in activities of daily living (KATZ-6-ADL > 0) (%)	38.6	41.4	41.8	42.1	41.7
Missing	7.3	5.9	3.4	4.6	4.7
Known with osteoporosis (%)	11.2	11.3	9.8	9.6	10.1
Missing	12.0	6.6	13.7	17.4	14.0
ASA score III, IV or IV (%)	54.2	54.8	55.3	57.4	56.1
Missing	7.4	3.1	5.8	2.3	3.8
Risk of malnutrition (%)					
No risk (SNAQ 0 or MUST 0)	77.1	80.4	81.3	80.5	80.5
Medium risk (SNAQ 1–2 or MUST 1)	4.9	3.6	3.7	3.6	3.8
High risk (SNAQ ≥ 3, MUST ≥ 2)	7.2	8.8	9.4	10.0	9.4
Missing	10.8	7.2	5.6	5.8	6.3
Patient outcomes					
Time on emergency ward in minutes (median [IQR])	170 [120, 230]	143 [105, 187]	151 [112, 200]	172 [130, 225]	161 [120, 212]
Missing	22.0	9.3	10.7	7.7	9.9
Surgery within 48 h (%)	91.2	90.6	93	92.7	92.3
Missing	2.2	3	1	1.5	1.7
Length of hospital stay in days (median [IQR])	5 [3, 8]	5 [3, 8]	5 [3, 8]	5 [3, 8]	5 [3, 8]
Missing	15.5	7.9	11.6	7.7	9.4
Orthogeriatric co-treatment (%)^b^	67.5	75	77.9	79.4	77.3
Missing	6.2	1.6	3.4	1	2.2
In-hospital mortality (%)	2	1.8	2.2	2.6	2.3
Missing	0.2	0.9	0.2	0.2	0.3
30 days mortality (%)^c^	4.8	5	5.7	5.8	5.6
Missing	0	0	0	0	0

*IQR* Interquartile Range, *KATZ-6 ADL* KATZ Index of Activities of Daily Living [16], *ASA score* American Society of Anesthesiologist physical status classification [17], *SNAQ* Short Nutritional Assessment Questionnaire [18], *MUST* Malnutrition Universal Screening Tool [19]

^a^Divided into quartiles of all hospitals’ annual volume: Low: < 122 patients per year; mid–low: 122–202 patients per year; mid–high: 203–322 patients per year; high:  > 322 patients per year

^b^Reported for patients aged 70 and older: percentages of respectively 2393, 6558, 10,103, 16,002 and total of 35,056 patients

^c^Reported for patients with Vektisdata available: percentages of respectively 2898, 7937, 11,824, 18,334 and total of 40,993 patients

**Fig. 2:**
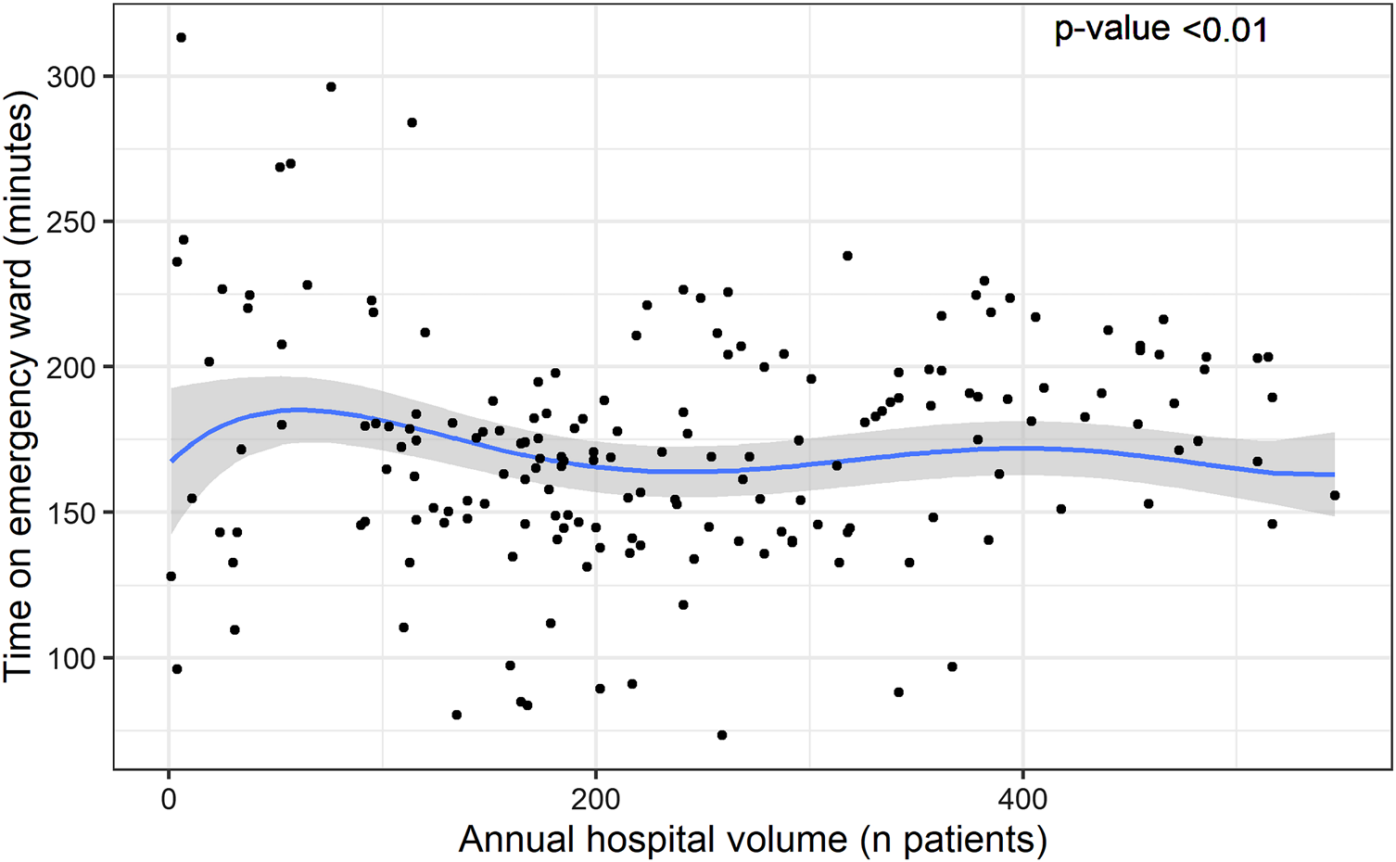
Multi-level fifth-degree polynomial regression model of time on emergency ward and annual hospital volume. Each dot represents the mean time on the emergency ward for a specific hospital in a specific calendar year

There was wide variation in the mean time in the ED between hospitals (range 86–280 min), regardless of the annual patient volume. In the mixed-effects polynomial regression model, the modeled time in the ED fluctuated between 161 and 181 min, with the widest variation between lower-volume hospitals. This association was statistically significant (*p* < 0.01) (Fig. 2).

**Fig. 3:**
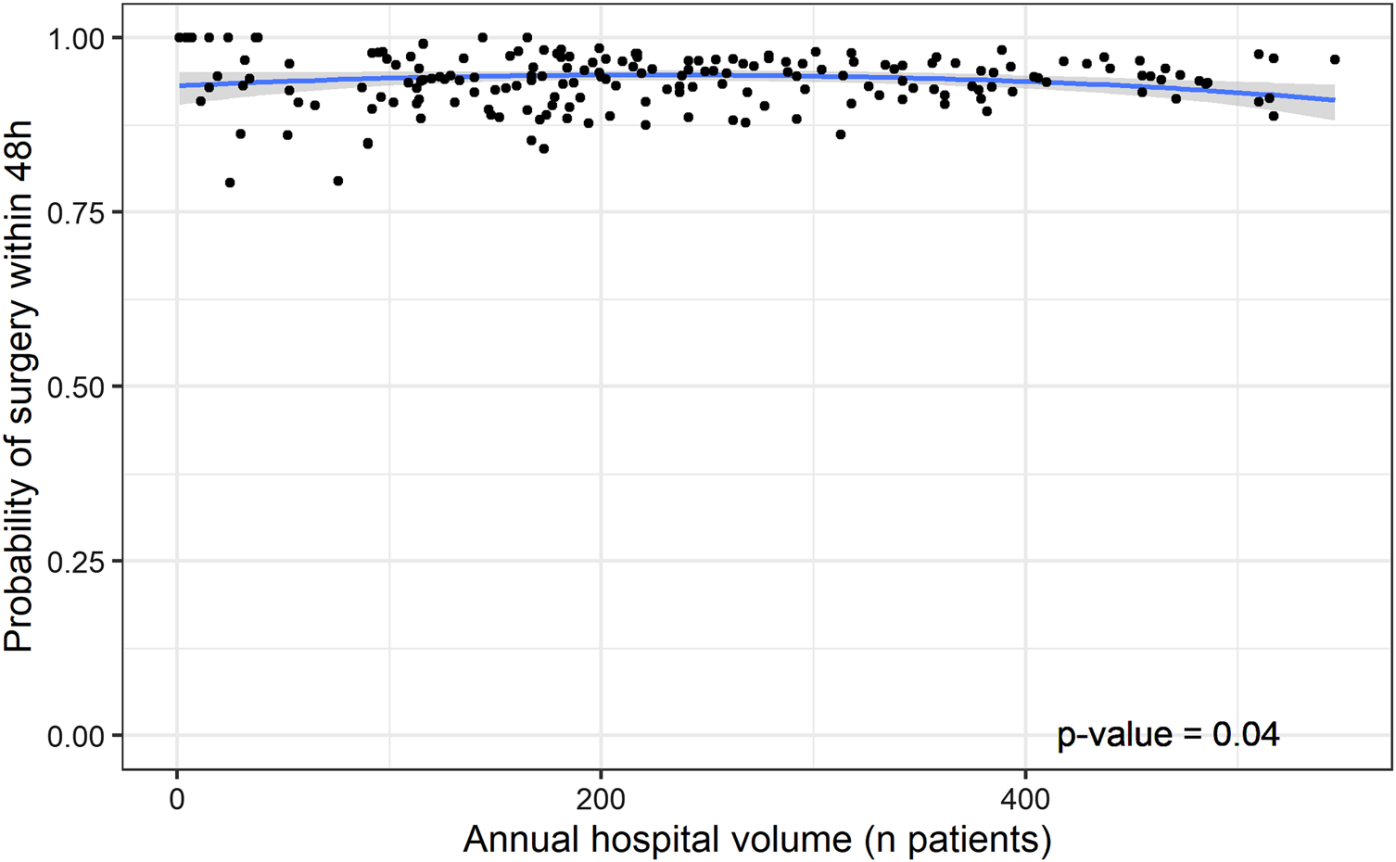
Multi-level second-degree polynomial regression model of probability of surgery within 48 h after presentation and annual hospital volume. Each dot represents the probability of surgery within 48 h for a specific hospital in a specific calendar year

The probability of surgery within 48 h was higher than 0.79 for all hospitals. The modeled probability of surgery within 48 h was stable at 0.94 up to an annual volume of 224 patients, while higher-volume hospitals showed a decrease to 0.91. There was a statistically significant association between the probability of surgery within 48 h and hospital volume (*p* = 0.04; Fig. 3).

**Fig. 4:**
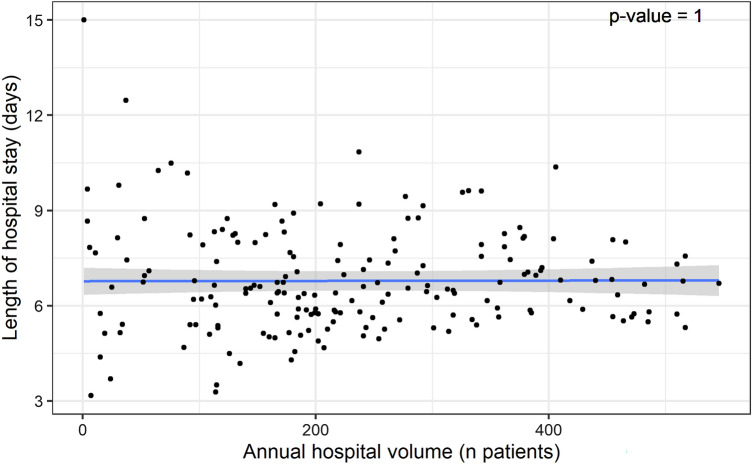
Multi-level first-degree polynomial regression model of length of hospital stay and annual hospital volume. Each dot represents the mean length of hospital stay for a specific hospital in a specific calendar year

The mean HLOS varied widely between the hospitals, especially for the lower-volume hospitals. However, no association between HLOS and hospital volume was found (*p* = 1; Fig. 4).

**Fig. 5:**
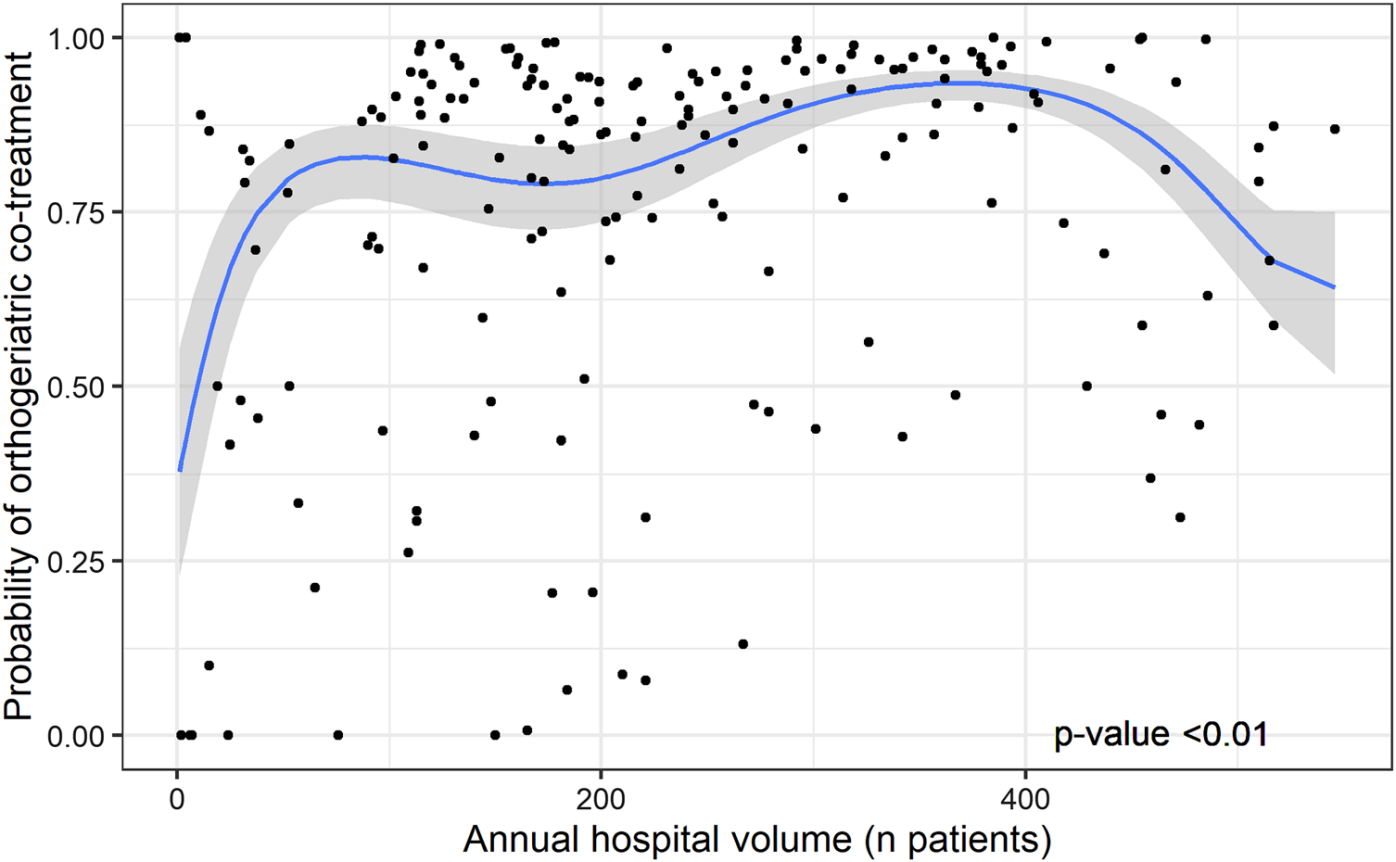
Multi-level fifth-degree polynomial regression model of probability of orthogeriatric co-treatment and annual hospital volume. Each dot represents the probability of orthogeriatric co-treatment for a specific hospital in a specific calendar year

For hospitals with annual volumes of up to 200 patients, the probability of orthogeriatric co-treatment varied between 0 and 100%. In the mixed-effects model, the probability of orthogeriatric co-treatment was low in lower-volume hospitals and was especially low in-hospital volumes between 0 and 100 patients. This probability increased when annual hospital volume increased to 367 patients per year (estimated probability of 0.93). A further increase in annual hospital volume was associated with a decreasing probability of orthogeriatric co-treatment (*p* < 0.01; Fig. 5).

**Fig. 6:**
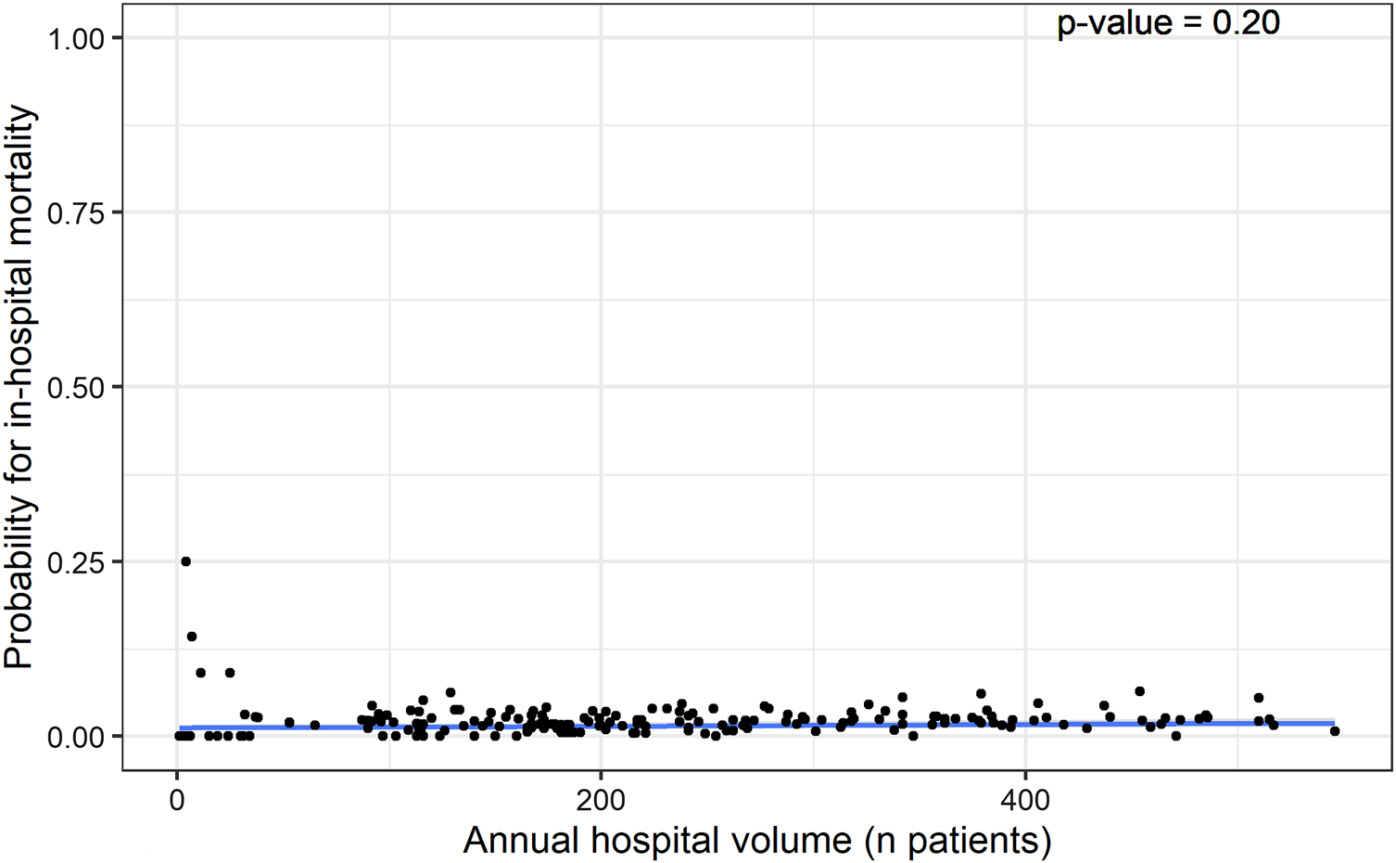
Multi-level first-degree polynomial regression model of probability of case-mix adjusted in-hospital mortality* and annual hospital volume. * Reference categories used were female gender, left-sided fracture, trochanteric AO-A2 fracture type, mobile outdoors with two aids or frame, independent in daily living activities, ASA score 3, 4 or 5, and no risk of malnutrition

**Fig. 7:**
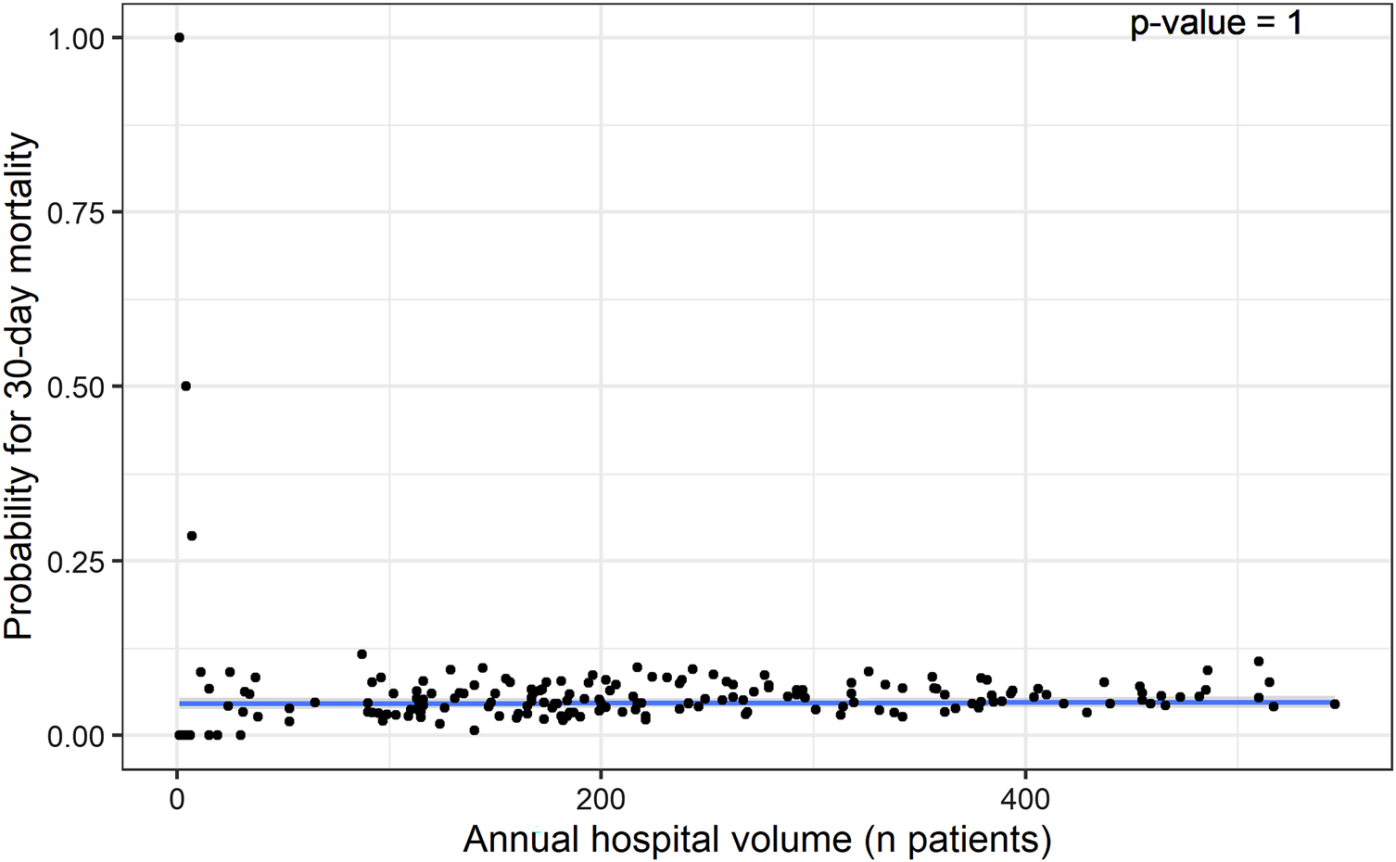
Multi-level polynomial regression model of probability of case-mix adjusted 30 days mortality* and annual hospital volume. *Reference categories used were female gender, left-sided fracture, trochanteric AO-A2 fracture type, mobile outdoors with two aids or frame, independent in daily living activities, ASA score 3, 4 or 5, and no risk of malnutrition

The case-mix adjusted models for both in-hospital and 30 days mortality showed no statistically significant association with hospital volume (*p* = 0.20 and *p* = 1, respectively; Figs. 6 and 7).

## Discussion

This study aimed to analyze the hospital volume effect on quality of hip fracture care in several domains: turnaround times, orthogeriatric co-treatment, and case-mix adjusted in-hospital and 30 days mortality. We found a significant and relevant relation between annual hospital volume and orthogeriatric co-treatment. Although statistically significant, models showed no clinically meaningful association between hospital volume and time in the ED and time to surgery within 48 h. No statistically significant effect of hospital volume was found for HLOS, and in-hospital and 30 days mortality. There seemingly is a wider variance in scores on processes and outcomes in lower-volume centers; however, this is likely explained by their smaller sample sizes.

Several generally accepted explanations exist for a positive effect of high volume on processes and outcomes. First, the ‘practice makes perfect’ principle; operating on higher patient numbers is assumed to make surgeons better at it, likely decreasing the risk of surgery-related complications. High patient volumes may not only affect the performance of individual surgeons; treating more patients may also affect processes and outcomes on institutional levels. Higher institutional volumes may allow for increased workflow, more homogeneity in treatment, better resource utilization and specialization of personnel [24]. A positive volume–outcome effect may also result from ‘selective referral’: a vicious circle in which high-performing hospitals increasingly receive more patients and gain more experience [12]. Examples of organizational benefits of high volume in hip fracture care are specific timeslots for hip fracture surgery, dedicated hip fracture treatment teams, specialized wards and implementation of evidence-based hip fracture care pathways [25]. Alternatively, higher volumes might lead to suboptimal quality of care and can negatively affect processes and outcomes, if high patient volumes lead to greater workloads than the organizational structure can handle.

The most striking result of our study is the relationship between hospital volume and orthogeriatric co-treatment. The probability of receiving orthogeriatric co-treatment increased with higher volumes, up to 367 patients per year. This is in line with the study by Shabani et al., who also found that higher-volume hospitals scored better on several preoperative medical assessments [9]. It is plausible that these hospitals are more likely dedicated hip fracture centers, with an orthogeriatric ward or a dedicated hip fracture team allowing co-treatment by a geriatrician. In our study, the probability of receiving orthogeriatric co-treatment decreased in hospitals treating over 367 patients per year. Possibly, under-capacity of staffing limits the relationship between hospital volume and this quality of care indicator.On the other hand, a more differentiated approach toward orthogeriatric co-treatment in higher-volume hospitals, based on extensive experience, may lead to a more selective deployment of medical specialist. More research is needed to elucidate these findings.

One could hypothesize that turnaround times are positively affected by the aforementioned organizational benefits of higher hospital volumes. However, the findings in our study do not clearly substantiate this hypothesis. Although we found statistically significant associations between patient volume and both time spent in the ED and surgery within 48 h, the clinical relevance of this finding is questionable. For time in the ED, the polynomial spline fluctuated, especially for lower volumes with a wider confidence interval, but did not show a trend toward increasing or decreasing turnaround times with the increase of volume. For surgery within 48 h, the modeled probability changed only by 3% (between 91 and 94%).The effect of hospital volume on turnaround times in the ED was only studied earlier by Shabani et al., who did not find an effect of volume on time to admission to an orthopedic ward, nor on the HLOS [9]. The latter finding corresponds with our study in which HLOS was not associated with hospital volume. The absence of this relation in our study contradicts most previous studies included in two reviews that both found that patients treated in low-volume centers had longer HLOS. However, these reviews and meta-analyses were limited by the various volume thresholds [10, 26]. Another striking finding is the relatively high percentage of patients operated on within 48 h. The time to surgery was used as an obligatory quality indicator for the Dutch Health Care Inspectorate in the past few years, which may have led to a shortening of the time to surgery, regardless of the hospital volume. We believe this to be a promising finding that underlines the expediency of hip fracture audits.

We did not find a relationship between hospital volume and case-mix corrected in-hospital and 30 days mortality. Wiegers et al. published a review analyzing over 2 million patients in 2019, including a meta-analysis. Ten out of twenty studies reported no hospital volume effect on in-hospital mortality, eight studies reported lower mortality in high-volume centers (threshold of > 170 patients/year), and two reported lower mortality in low-volume centers. The meta-analysis did not show an overall statistically significant association between hospital volume and in-hospital mortality. However, we believe that this nonsignificant overall result, again, is due to the wide variance in thresholds used [10]. Contradictorily, a scoping review of studies covering twelve different surgical specialties by Levaillant et al. reported that 86.2% of the studies included showed a significantly positive effect of higher hospital volume on mortality. The absence of a volume–outcome relation for mortality in hip fractures and the apparent presence of this volume–outcome relation in other surgical specialties might be explained by differences in the complexity of the surgical interventions [8].

This is the first study in which a large cohort of hip fractures is used to analyze the volume effect on multiple processes and outcomes on a patient level, using volume as a continuous parameter. Another strength of this study is the use of an extensive case-mix model in the analysis of mortality outcomes. The main limitation of this study concerns the use of registry data, of which the researchers could not validate the quality. Due to limitations in the number of complete years of registration, we could not perform internal validation of the models.

Our study has implications for the debate on centralization of hip fracture care. Our results do not justify the centralization of hip fracture services for the sole purpose of improving the quality of care provided. Needless to say, this conclusion holds true only for the process and mortality outcome parameters tested in this study. Future studies could analyze surgical complications and functional outcomes and evaluate the effect of provider volume. Also, future studies could include a continuous volume–value analysis, as the effect of higher volume or centralization may not merely impact the quality of care but also affect hip fracture care costs [11]. We believe orthogeriatric co-treatment to be impacted by hospital volume in the Netherlands and should therefore be further investigated. Instead of centralizing care and thereby withholding patients geographically accessible care, it would be better to share best practices and enhance collaborations between hospitals to improve the quality of hip fracture care on a national level.

### Conclusion

This study showed that hospital volume does not have a clinically relevant effect on turnaround times, nor does it affect in-hospital and 30 days mortality. However, orthogeriatric co-treatment within the nationwide hip fracture registry in the Netherlands seems to be provided more often in higher-volume hospital with a maximum of 367 patients and should be further analyzed. Although our findings may be relevant in the centralization debate, additional analysis of complications and functional outcomes treating volume as a continuous parameter is indicated to draw final conclusions on the effect of hospital volume on the quality of hip fracture care.

## Data Availability

The data that support the findings of this study are available on request through the Dutch Institute of Clinical Auditing. Restrictions apply to the availability of these data, which were used under license for this study.
